# Fast and accurate interpretation of workload classification model

**DOI:** 10.1371/journal.pone.0282595

**Published:** 2023-03-06

**Authors:** Sooyeon Shim, Doyeon Kim, Jun-Gi Jang, Suhyun Chae, Jeeyong Lee, U. Kang

**Affiliations:** 1 Seoul National University, Seoul, Republic of Korea; 2 Samsung Electronics, Suwon, Republic of Korea; TU Wien: Technische Universitat Wien, AUSTRIA

## Abstract

How can we interpret predictions of a workload classification model? A workload is a sequence of operations executed in DRAM, where each operation contains a command and an address. Classifying a given sequence into a correct workload type is important for verifying the quality of DRAM. Although a previous model achieves a reasonable accuracy on workload classification, it is challenging to interpret the prediction results since it is a black box model. A promising direction is to exploit interpretation models which compute the amount of attribution each feature gives to the prediction. However, none of the existing interpretable models are tailored for workload classification. The main challenges to be addressed are to 1) provide interpretable features for further improving interpretability, 2) measure the similarity of features for constructing the interpretable super features, and 3) provide consistent interpretations over all instances. In this paper, we propose INFO (INterpretable model For wOrkload classification), a model-agnostic interpretable model which analyzes workload classification results. INFO provides interpretable results while producing accurate predictions. We design super features to enhance interpretability by hierarchically clustering original features used for the classifier. To generate the super features, we define and measure the interpretability-friendly similarity, a variant of Jaccard similarity between original features. Then, INFO globally explains the workload classification model by generalizing super features over all instances. Experiments show that INFO provides intuitive interpretations which are faithful to the original non-interpretable model. INFO also shows up to 2.0× faster running time than the competitor while having comparable accuracies for real-world workload datasets.

## Introduction

*How can we provide accurate and fast interpretations for a workload classification model?* A workload classification task is to classify a given subsequence, which contains a series of tuples of commands and locations for memory accesses, into a workload type that generates the sequence. As new AI-based applications including self-driving cars and mobile applications emerge, the demands for memory devices like DRAM (Dynamic Random Access Memory) rapidly grow. Since the manufacturers require high-quality memory devices, verifying and improving the quality of DRAM is a crucial task in the real world. An accurate workload classification model helps improve the quality of DRAM. In utilizing a workload classifier, it greatly helps to know why a workload subsequence is classified to a specific class, or which part leads to the misclassification.

There are 5 heterogeneous fields within an operation: command, rank, bank group, bank, and address. The previous state-of-the-art model Acorn [1] generates 3 types of features using the 5 fields: CMD, bank-level, and cell-level features. CMD features capture sequential information of the command field. Bank-level and cell-level features are related to address-related fields, i.e., rank, bank group, bank, and address fields. Bank-level feature vectors describe the number of accesses for each bank, where the address field refers to a row or column address of a specific bank. Cell-level features map an address into a memory region inside banks, to capture spatial information within banks.

An accurate, but black-box workload classification model fails to provide the interpretation for the prediction of a given subsequence. For example, it does not explain what features mainly affect the classification result, and why the subsequence is classified into a class. Therefore, we need to exploit an interpretable model to understand the results of subsequence classification. Ribeiro et al. [2] propose LIME which provides interpretation for an instance. Since features used for a neural network cannot be understandable to humans, LIME interprets the prediction for an image using super-pixels obtained by image segmentation. However, LIME cannot give explanations over a workload classifier because super-pixels are limited only to image interpretation. Thus, we need to design appropriate super features for workload classification. Another limitation of LIME is its local interpretability which needs to learn a new model to explain a new instance. Such a locally interpretable model entails a long running time which limits its use for workload classification.

**Fig 1:**
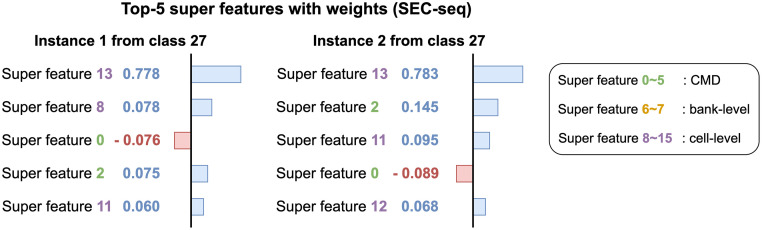
Interpretation of two instances for class 27 from the SEC-seq dataset. Both instances are correctly classified as class 27. Three types of super features are denoted as green, yellow, and purple colors, respectively. Note that cell-level super features, especially the super feature 13, influence the predictions the most while super feature 0 commonly gives a negative impact. In other words, INFO accurately predicts labels of input instances as the class 27 since the instances have a unique pattern of accessing a specific memory region related to super feature 13.

In this paper, we propose INFO (INterpretable model For wOrkload classification), a fast and accurate model for workload classification. We carefully design super features for workload classification. For effective clustering, we propose an interpretability-friendly similarity measure between the original features based on a variant of Jaccard similarity. We also exploit the locality of the bank-level and cell-level features. INFO provides global explanations using super features commonly defined for every instance. Since we use the same super features over all instances, we provide consistent interpretations such as analyzing what super features mainly affect the predictions for subsequences in the same class. To the best of our knowledge, the proposed method is the first work that explains the classification results of workloads. Fig 1 shows an example of explained results provided by INFO. INFO gives a global interpretation, which means that the same super features are used to explain different instances. Thus we know that cell-level features clustered as a super feature 13 capture the most crucial patterns of class 27 leading to an accurate classification. Experimental results demonstrate that INFO gives interpretations that are faithful to the non-interpretable workload classifier.

We summarize our main contributions as follows:

- **Method**. We propose INFO, a globally explainable model which gives fast and accurate interpretations for workload classification.
- **Interpretation**. We enhance the interpretability of the proposed method by clustering the original features based on the hierarchical characteristics of workload data.
- **Experiments**. We show the interpretation results and prove the effectiveness of the proposed model. INFO achieves up to 2.0× faster running time than the competitor while having a comparable accuracy on real-world workload data.

The rest of the paper is organized as follows. We provide preliminaries, our proposed method, and experimental results in order. Then, we present related works and the conclusion. The codes and data used for INFO are available at https://github.com/snudatalab/INFO.

## Preliminaries

In this section, we describe the preliminaries and our problem definition. Table 1 describes the symbols used in this paper.

**Table 1:** Description of symbols.

Symbol	Description
*W*	A workload sequence of size *l* × 5
*w*	A workload subsequence of size 100, 000 × 5
*f*	a trained workload classification model
*g*	an interpretable model
**x**	a feature vector for classification
**x**′	a vector with super features for interpretation
**z**′	randomly generated vector from **x**′
**z**	recovered vector of **z**′ to the original feature space
Z	a set of (**z**′, *f*(**z**)) for training the model *g*

### Workload sequence

We define a *workload sequence* produced by a DRAM controller unit during a program execution referring to [1].

**Definition 1** (Workload Sequence). *A workload sequence* $W\in R^{l\times 5}$ *is a matrix where l is the length of a workload and* 5 *is the number of fields*.

- *Command field contains a series of commands where each command is mapped to a number, e.g*., 1 *refers to ACT command*.
- *Rank field is an index of a rank, the highest level of DRAM components*.
- *Bank group field refers to the bank group number within a rank*.
- *Bank field contains an index of a bank in a bank group*.
- *Address field represents a row or column address within a bank*.

We define the concept of *workload subsequence* referring to [1] and use it as an instance of the proposed method.

**Definition 2** (Workload Subsequence). *A workload subsequence* $w\in R^{100,000\times 5}$ *is a sub matrix of W where its row length is fixed as* 100, 000.

### Workload classification

The task of workload classification aims to classify a given subsequence into a workload type that generates it. Each workload contains a sequence of operations where each operation contains 5 heterogeneous fields. The previous state-of-the-art method Acorn [1] performs a workload classification task with carefully designed features including CMD feature, bank-level feature, and cell-level feature. The final feature vector for classification is a concatenation of CMD, bank-level, and cell-level feature vectors. Acorn predicts the label of a subsequence by feeding its feature vector into a neural network. Although Acorn achieves high accuracy for the workload classification, it does not give an easy interpretation of its result because it is a complicated deep learning model. In this work, we additionally provide interpretability for the workload classification model.

### LIME: Model-agnostic interpretable model

LIME [2] is a model-agnostic interpretable model which is locally faithful to the pre-trained model. Given an instance and a trained model, LIME aims to interpret the prediction of a given instance. For an image classification task, LIME exploits super-pixels which are obtained through image segmentation where each segment corresponds to a super-pixel. LIME trains a linear model by sampling instances in the vicinity of a given instance. Note that features used for a linear model are super-pixels. The trained weights of a linear model correspond to the contributions super-pixels give to the prediction. LIME is not ready for workload classification for the following two reasons. First, it is not clear how to construct the interpretable features for workload classification. Second, LIME provides local interpretation which gives inconsistent interpretations when we compare the results of many instances. We address the two challenges with the proposed INFO.

### Problem definition

We introduce the formal problem definition as follows:

**Problem 1** (Interpretation for Workload Classification). ***Given*** *a subsequence and a trained model that classifies the subsequence*, ***find*** *an interpretable model over the subsequence, which is faithful to the trained model*.

## Proposed method

In this section, we propose INFO for interpreting predictions of a workload classification model. The challenges to be addressed are to 1) provide intuitive and interpretable features, 2) measure the similarity of raw features for constructing the interpretable features, and 3) provide consistent interpretations over all instances. To tackle the above challenges, our ideas are to 1) carefully design super features for workloads, 2) compute the interpretability-friendly similarity, and 3) provide consistent interpretations with global super features over all instances.

Algorithm 1 shows the overall process of INFO. Before interpreting a given test instance, we find super features for three types of feature vectors: CMD, bank-level, and cell-level features. Given a test instance, a set of super features, and a trained classification model *f*, we first construct a binary vector of super features (line 1 in Algorithm 1). Then, we construct a dataset based on the binary vector and the test instance (line 2 in Algorithm 1), and learn an interpretable model that approximates the given classification model *f* (line 3 in Algorithm 1). Finally, we interpret the classification results using the weights of the interpretable model, which represent the amount of attribution of super features.

**Algorithm 1**: INFO: Interpretable Model for Workload Classification

**Input**: A test instance **x**, a set S of super features, a trained classification model *f*

**Output**: Weights of an interpretable model *g*

 1: **Defining super feature vector**. Define $x^{\prime }\in R^{d^{\prime }}$ where *d*′ refers to the number of super features in S. **x**^**′**^ is a binary vector full of 1s which indicates that all super features appear at the given subsequence.

 2: **Data Construction**. Randomly sample $z^{\prime }\in R^{d^{\prime }}$ from **x**′. Construct a dataset Z for the model *g* as pairs of (**z**′, *f*(**z**)) where $z\in R^{d}$ is a vector that recovers **z**′ to the original feature space. If *i*-th element of **z**′ is 1, **z** recovers the values of features within *i*-th super feature. Otherwise, the values remain as 0.

 3: **Training the model**. Train the model *g* using the training set Z by minimizing *L*(*f*, *g*, *π*_**x**_) in Proposed Method section that makes *g* approximate the model *f*. Return the weights of the model *g* which are the attribution values each super feature gives to the classification result of the given instance.

In the following, we first describe how to design super features by clustering raw features used in a workload classification model. We then propose INFO which gives global interpretations using the same super features over all instances.

### Construction of super features

Our objective is to find interpretable super features which reflect original features used in a classification model. Directly using the original features to a local interpretable model degrades the interpretability due to their large number and insufficient information. For example, in an image classification task, interpretation with pixels corresponding to features is more difficult than that with super-pixels which are segmentation results of the image since the super-pixels are more intuitive than the pixels. To improve the interpretability in the workload classification task, we exploit super features corresponding to the super-pixels in images. Our approach is to cluster original features by exploiting their characteristics, and use the clusters as super features.

**Fig 2:**
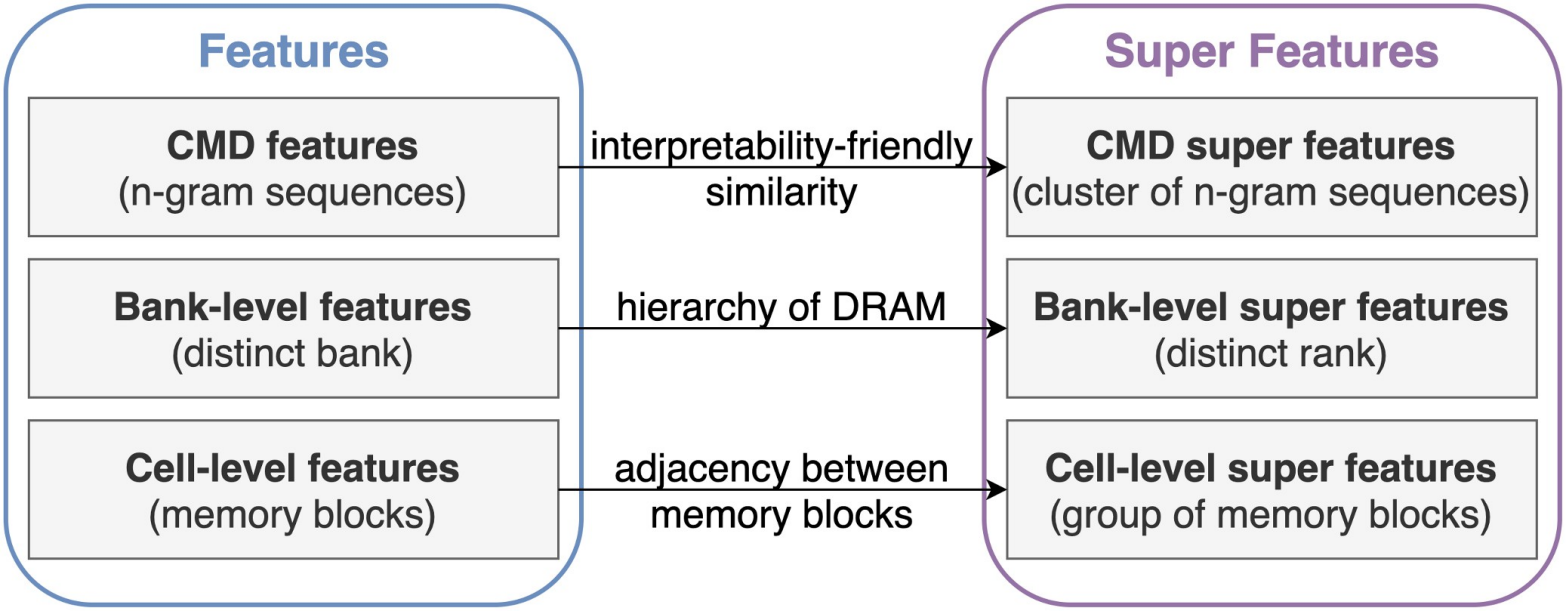
Overall process of generating super features. We define three different types of super features for workload classification. Super features are designed to capture the similarity between the original features, and are commonly applied to every workload subsequence.

To obtain super features, we independently perform clustering for the three types of feature vectors in workload classification: CMD, bank-level, and cell-level features. CMD features contain sequential information within a command field. Bank-level and cell-level features are extracted from address-related fields, i.e., rank, bank group, bank, and address fields, capturing spatial information within them. Fig 2 summarizes the process of generating super features for workload classification.

#### CMD super features

Our goal is to find super features from n-gram CMD features. Although the most frequent n-gram sequences are selected as features for workload classification, they still have redundant sequential patterns. For example, n-gram sequences 33113 and 13133 include common sequences 33 and 13. Therefore, we need to construct super features of CMD features by clustering similar n-gram sequences, and use them for interpreting a prediction result of workload classification. To achieve it, we propose an interpretability-friendly similarity to measure the similarity between n-gram sequences.

**Fig 3:**
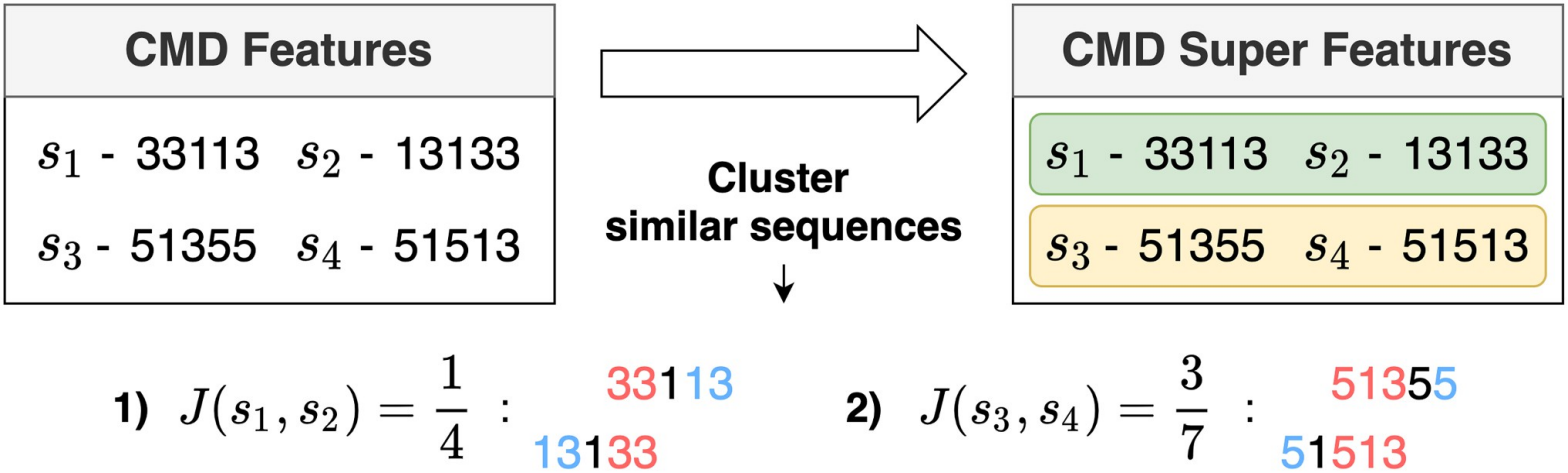
Illustration for generating CMD super features. The first super feature includes sequences *s*_1_ and *s*_2_, and the second super feature includes sequences *s*_3_ and *s*_4_.

Before clustering n-gram sequences, we define an interpretability-friendly similarity to group the CMD features. We propose a variant of Jaccard similarity to measure the similarity between two n-gram sequences. Given two n-gram sequences *s*_1_ and *s*_2_, we define their similarity *J*(*s*_1_, *s*_2_) as follows:

$$\begin{array}{c} J\left(s_{1},s_{2}\right)=\frac{\text{max}(l_{s_{1},s_{2}},l_{s_{2},s_{1}})}{l_{s_{1}}+l_{s_{2}}-\text{max}\left(l_{s_{1},s_{2}},l_{s_{2},s_{1}}\right)} \end{array}$$ (1)

where $l_{s_{1}}$ is the length of a sequence *s*_1_ and $l_{s_{1},s_{2}}$ is the length of the common subsequence between the front of *s*_1_ and the end of *s*_2_. Fig 3 gives an example of generating CMD super features by calculating the similarity.

After measuring the similarity for all n-gram sequences, the next step is to perform hierarchical clustering based on the similarity. Treating each n-gram sequence as its cluster, we identify the most similar pair of clusters and fuse these two clusters. The similarity between two clusters is measured using the average similarity between the elements in the two clusters. Then, we repeat the previous process for the remaining clusters until the desired number of clusters remains. Finally, we use the clustering results as CMD super features.

#### Bank-level super features

**Fig 4:**
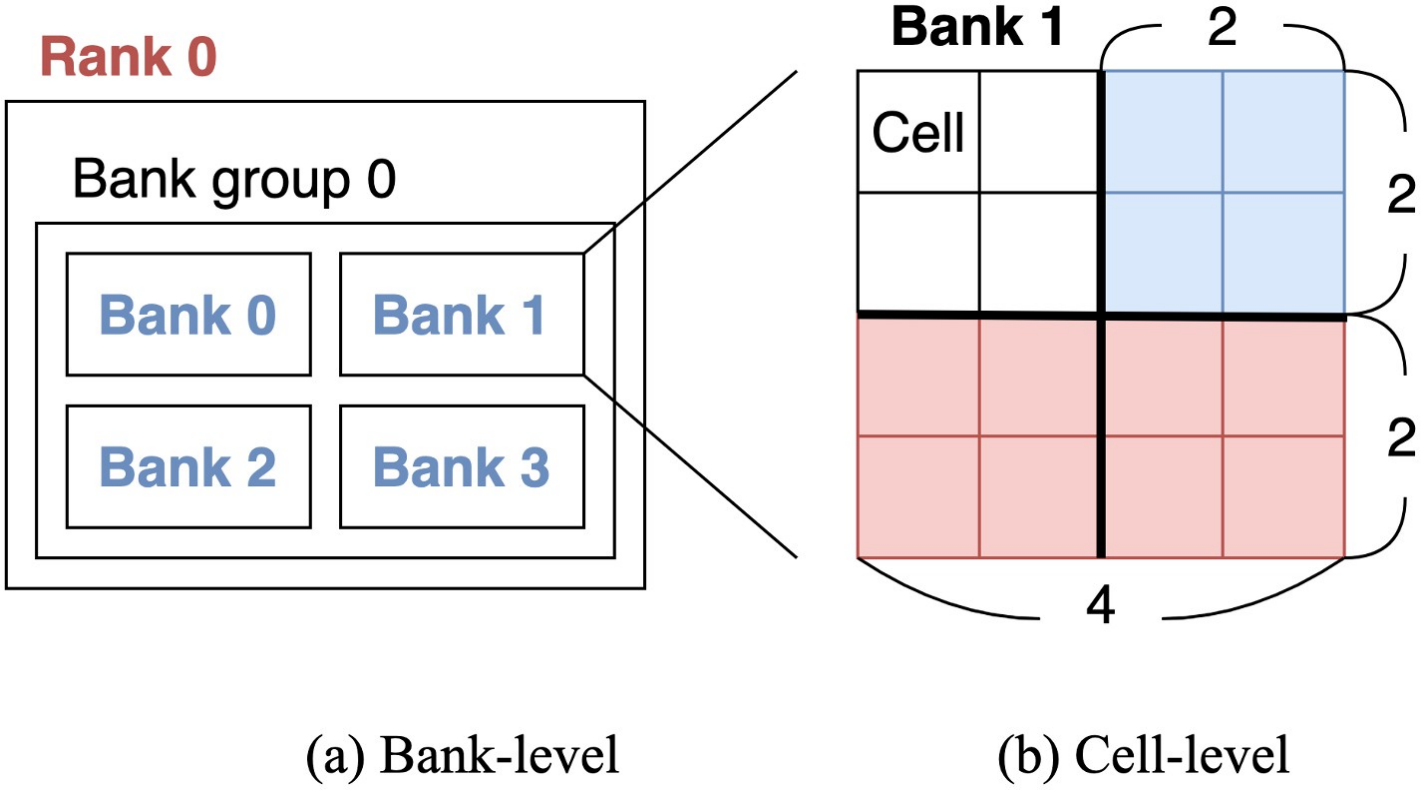
An example of constructing bank-level and cell-level super features. (a) shows a bank-level super feature extracted from rank, bank group, and bank fields. (b) shows a cell-level super feature including the address field. Features and super features are denoted as blue and red colors, respectively.

Our goal is to obtain super features from bank-level features which include rank, bank group, and bank fields. As discussed in the Preliminaries section, the rank field is at the highest level among the bank-level features. Therefore, we use the rank as a bank-level super feature based on the hierarchical structure. Fig 4(a) shows an example of DRAM with one rank, one bank group within a rank, and four banks within a bank group. In this example, the four banks correspond to one super feature, Rank 0.

#### Cell-level super features

Our goal is to find super features for cell-level features. As for cell-level super features, we group adjacent memory blocks by considering the locality within a bank. We define each group of memory blocks as a cell-level super feature. Fig 4(b) illustrates an example of cell-level super features in Bank 1 of size 4 × 4. There are four memory blocks of size 2 × 2 colored blue. Then, we cluster two adjacent memory blocks and define a cell-level super feature of size 2 × 4 colored red.

### Locally interpretable model for workload classification

We aim to propose an interpretable model for workload classification utilizing super features.

As a naive approach, we can adopt a previous explanation model called LIME [2] directly to the workload classification model which provides interpretation over an instance. When applied to image classification task, LIME generates super-pixels for each image through image segmentation and provides interpretation over an image. LIME can be used to explain the workload classification result of an instance using super features.

**Fig 5:**
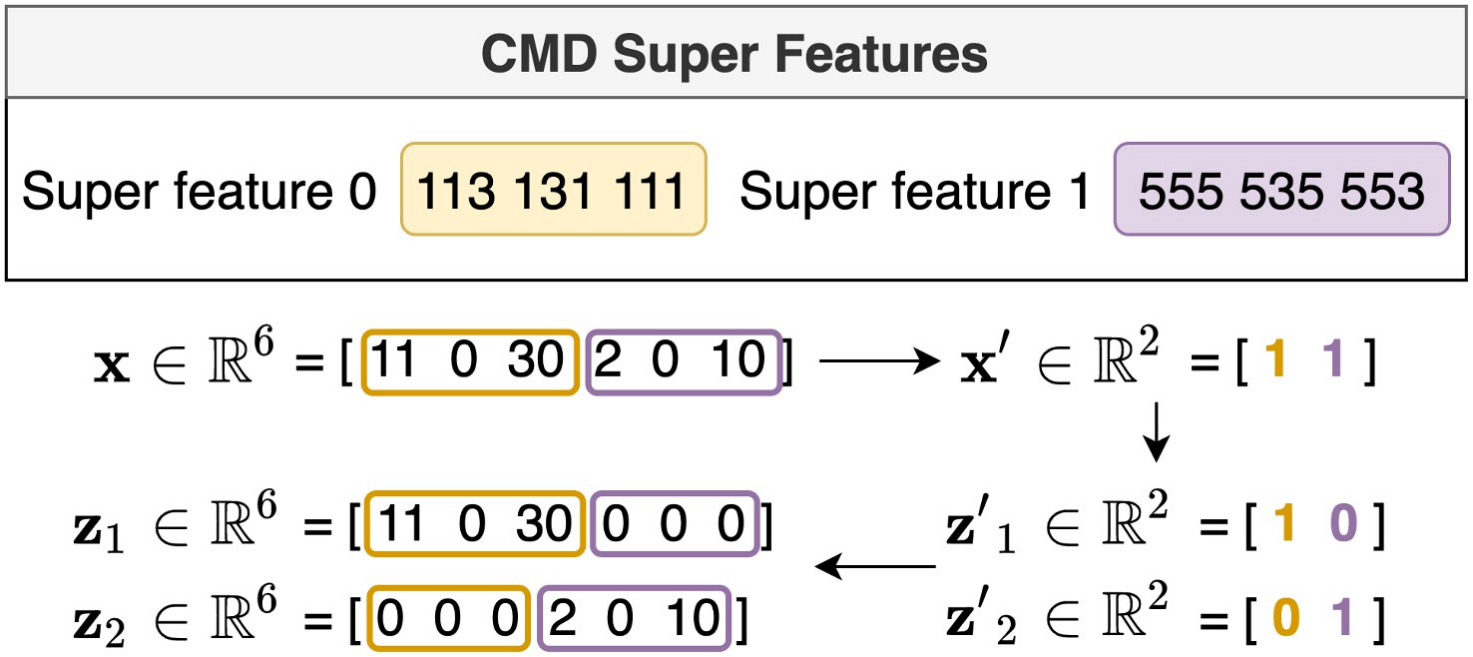
An example of constructing vectors for an interpretable model. An entry of a feature vector **x** denotes the number of occurrences of each n-gram sequence. For example, 11, 0, 30, 2, 0, and 10 are the numbers of occurrences of sequences 113, 131, 111, 555, 535, and 553 in an instance, respectively. **x**, **z**_1_, and **z**_2_ are defined in the original feature space while **x**′, **z**′_1_, and **z**′_2_ are represented using super features.

Given a feature vector of a subsequence, we need to convert it into a vector with super features for interpretation. We cluster n-gram sequences appearing in a given instance to generate CMD super features. Bank-level and cell-level super features are ranks and collections of memory blocks, respectively. We define a feature vector $x^{\prime }\in R^{d^{\prime }}$ for an interpretable model where *d*′ is the total number of super features. 1 and 0 in **x**′ indicate the presence and absence of the corresponding super feature in the given subsequence, respectively. For example, if a subsequence has accessed the first rank, the corresponding element of **x**′ is 1. We then train a linear model *g* which has interpretability in itself. We generate a training dataset Z for a linear model by randomly sampling the binary vectors $z^{\prime }\in R^{d^{\prime }}$ from **x**′. The label of **z**′ is the prediction of the pre-trained model *f* denoted as *f*(**z**); $z\in R^{d}$ is a recovered vector of **z**′ where *d* equals the total number of features for workload classification. Fig 5 illustrates the process of constructing training data for an interpretable model *g*, using n-gram features. **x** is a feature vector for a workload classification model *f*, where each element of **x** denotes the number of occurrences of the corresponding n-gram sequence in a given instance. To be specific, 11, 0, 30, 2, 0, and 10 are the numbers of occurrences of sequences 113, 131, 111, 555, 535, and 553 in a given instance, respectively. We define a feature vector **x**′ for an interpretable model *g* using CMD super features. Since all super features have appeared in a given subsequence, all elements of **x**′ are 1. Two binary vectors **z**′_1_ and **z**′_2_ are randomly sampled from **x**′ and used to train the model *g*. In this example, two pairs of (**z**′, *f*(**z**)) are set as Z. The model *g* is trained to approximate the pre-trained model *f* by minimizing Eq (2).

$$\begin{array}{c} L\left(f,g,\pi_{x}\right)=\sum_{z,z^{\prime }\in Z}\pi_{x}\left(z\right){\left(f\left(z\right)-g\left(z^{\prime }\right)\right)}^{2} \end{array}$$ (2)

*π*_**x**_ = exp(−*D*(**x**, **z**)^2^/*σ*^2^) measures the similarity between **x** and **z** where *D*(⋅) denotes a distance function and *σ* is a kernel width. We provide explanations using weights of a linear model which implies the amount of attribution each super feature gives to the prediction.

### Globally interpretable model for workload classification

We propose INFO, a model-agnostic interpretable model for workload classification. INFO provides global super features for workloads and provides explanations over all instances by utilizing them. Unlike the locally interpretable model (e.g., LIME) which gives inconsistent interpretation over different instances, INFO gives consistent interpretation over all instances by generating global super features that are applied to the entire subsequence.

For CMD super features, we cluster all n-gram sequences used as CMD features, and define each group as a super feature. The intuition is that clustering the whole CMD features represents the workloads better than clustering a few n-gram sequences. Generating super features from all CMD features increases computational efficiency because the similarity between features is calculated once. In contrast, the naive approach, which is locally interpretable, should calculate the similarity of features for each test instance and thus requires a long running time. We utilize the rank field as a bank-level super feature based on the structure of DRAM. We compose cell-level super features by clustering the adjacent memory blocks. The process of generating vectors for super features and training an interpretable model *g* is the same as in the naive approach. Unlike the naive method, INFO explains which super feature contains the most representative patterns over different instances. Global super features applied to all subsequences enable the proposed method to give accurate and fast interpretations that are consistent over the classes.

## Experiments

In this section, we experimentally evaluate the performance of INFO. We answer the following questions:

Q1. **Performance**. How fast and accurately does INFO interpret predictions of a workload classification model?
Q2. **Interpretation**. Does INFO give intuitive interpretations that are faithful to a workload classification model?
Q3. **Clustering**. Are CMD super features well-clustered?

### Experimental setting

We construct all models using the Pytorch framework. All the models are trained and tested on a machine with GeForce GTX 1080 Ti GPU.

#### Dataset

We use two datasets which contain real-world workload sequences summarized in Table 2. There are 40 and 31 workloads in SEC-seq and Memtest86-seq datasets, respectively. The lengths of workload sequences are different in both datasets. Each workload corresponds to a class and workload subsequences of length 100, 000 are used as instances where a label is given as a workload that the subsequence belongs to.

**Table 2:** Datasets for a workload classification.

dataset	# of classes	input size	# of train	# of test
**SEC-seq** ^1^	40	1735	586,885	293,444
**Memtest86-seq** ^2^	31	1599	433,334	216,696

^1^ Private to a company.

^2^ https://github.com/snudatalab/INFO

#### Hyperparameter settings

We use three different lengths of n-grams: *n* = 7, 11, and 15. We set features for the CMD field as the collection of Top-25 n-grams selected from the entire workload. The size of the CMD feature vector corresponds to the total number of selected n-grams. The numbers of 7-gram, 11-gram, and 15-gram sequences used as CMD features in SEC-seq dataset are 154, 236, and 289, respectively. For Memtest86-seq dataset, the numbers of 7-gram, 11-gram, and 15-gram sequences chosen as CMD features are 132, 196, and 215, respectively. We cluster n-gram sequences into 6 groups and use each group as a CMD super feature. We use DRAM with 2 ranks, 4 bank groups within each rank, and 4 banks within each bank group. The total number of banks is 2 × 4 × 4 = 32, which is the size of a bank-level feature vector. As we define a bank-level super features as a rank, the number of bank-level super features is 2. For each bank, we divide it into 8 × 128 memory blocks of size 2^14^ × 8 to count the number of accesses per block. The length of a cell-level feature vector is 1, 024, which is equal to the number of memory blocks. We generate cell-level super features by horizontally combining adjacent blocks: we utilize 8 memory block units of size 2^14^ × 2^10^ as cell-level super features.

#### Evaluation metrics

We use two evaluation metrics: Top-1 accuracy and Top-3 accuracy. Both metrics are used to measure the fidelity of an interpretable model to a black box model. Top-1 accuracy (%) is equal to (*N*_*top*−1_/*N*) × 100 where *N*_*top*−1_ denotes the number of test instances that both a black box model and an interpretable model agreed in its class prediction. *N* is the number of test instances. Top-3 accuracy (%) is defined as (*N*_*top*−3_/*N*) × 100 where *N*_*top*−3_ is the number of test instances where the predicted class from a black box model is included in the highest 3 classes predicted from an interpretable model.

### Performance

We evaluate how faithful and fast INFO is, compared to LIME which is a locally interpretable model that generates super features individually for each test instance. We adopt LIME to our setting by generating super features for each subsequence as described in the Proposed Method section. In contrast, the proposed INFO generates a unified set of super features which further improves the interpretability. As mentioned in the Experimental setting section, we use Top-1 and Top-3 accuracies as evaluation metrics. Both metrics compare the classification result of a black box model and a linear model. Top-1 accuracy computes the ratio of test instances that the predicted classes of two models are the same. Top-3 accuracy computes the ratio of test instances that the class predicted from a black box model is within the highest 3 predicted classes from a linear model. In addition, we compare the running time of INFO and LIME.

**Fig 6:**
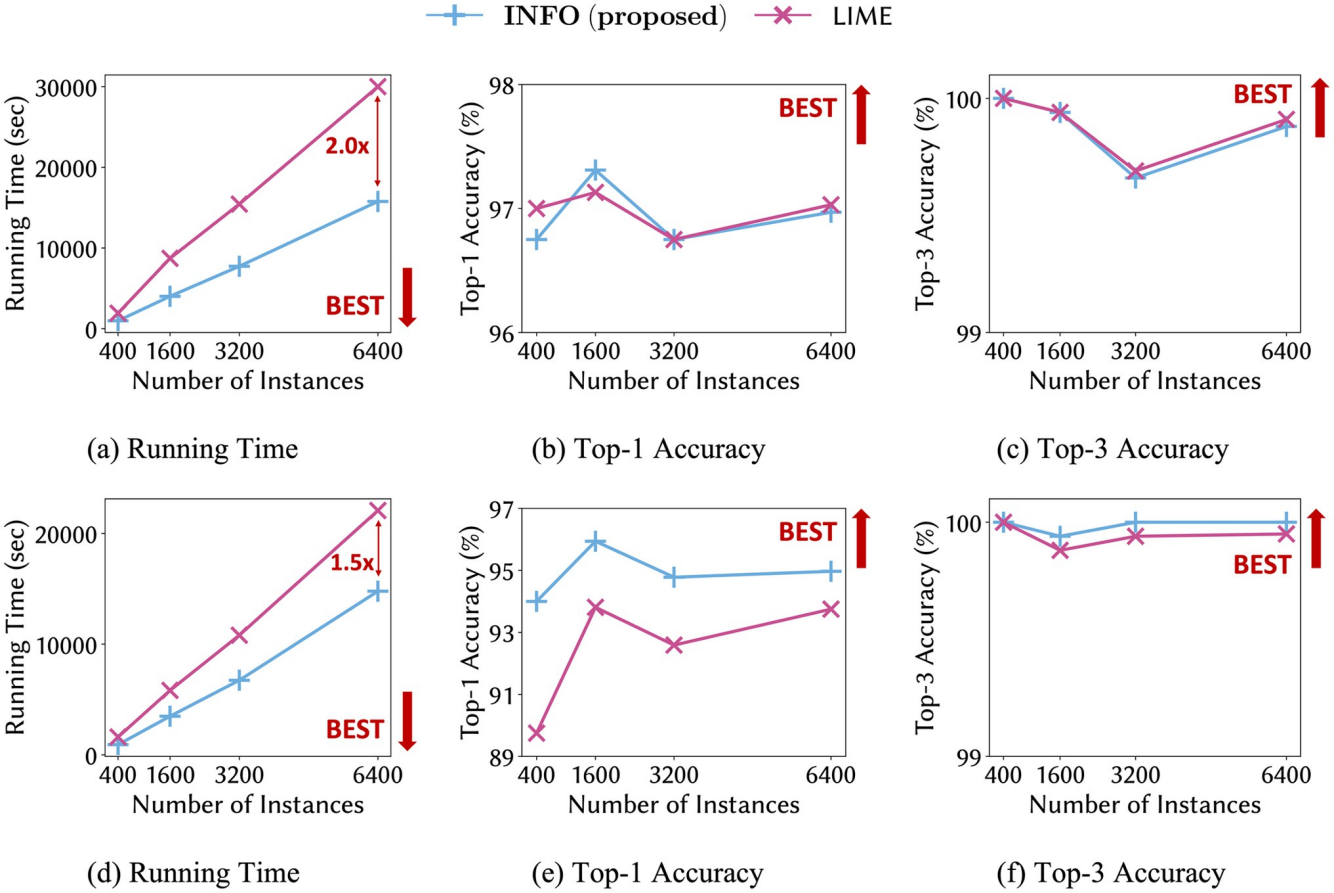
Running time and accuracy for INFO and LIME. (a-c) and (d-f) show performance on SEC-seq and Memtest86-seq datasets, respectively. INFO is up to 2.0× and 1.5× faster than LIME while having similar Top-1 and Top-3 accuracies for SEC-seq and Memtest86-seq datasets, respectively.

We measure the fidelity using 1, 000 test instances. To generate interpretable linear models for INFO and LIME, we create 10, 000 random samples from each instance and use them for training. We report the running time and the fidelity results of two datasets in Fig 6. INFO is up to 2.0× faster than LIME using the SEC-seq dataset. The performance gap becomes larger as the number of instances increases. For the Memtest86-seq dataset, INFO is also up to 1.5× faster and has higher Top-1 and Top-3 accuracies than LIME.

### Interpretation

**Fig 7:**
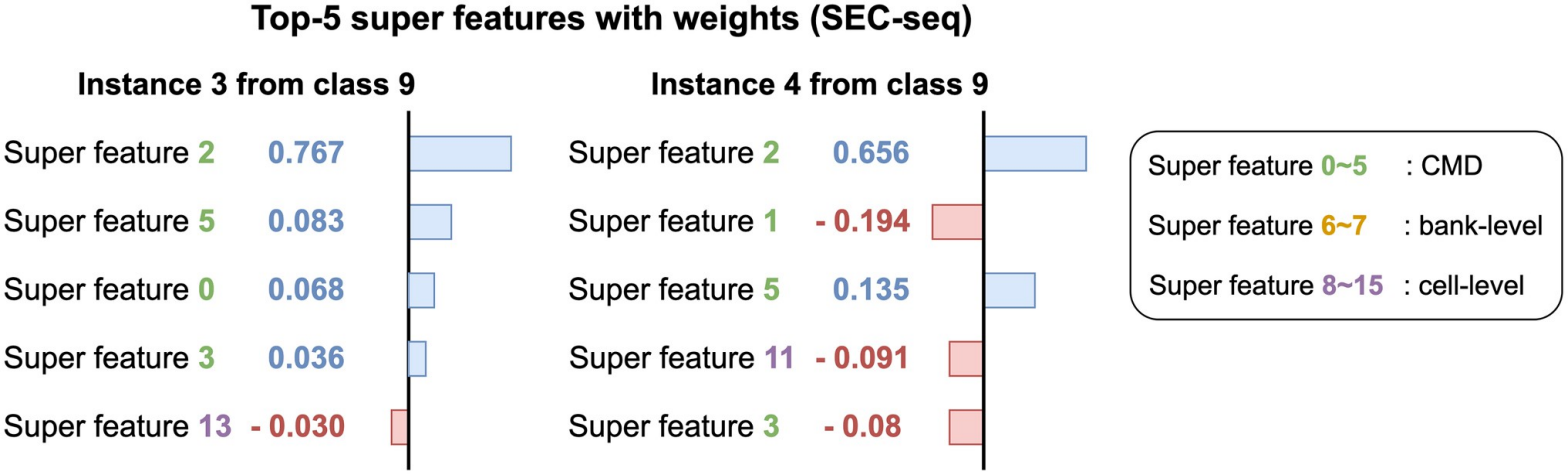
Interpretation of two instances for class 9 from SEC-seq dataset. Instance 3 is correctly classified but instance 4 is misclassified as class 8. Super feature 2 helps classify instances into class 9 while super features 1, 11, and 3 restrain the instance 4 from being classified into class 9.

We explain the prediction results of test instances using INFO. Fig 1 explains why two instances of the SEC-seq dataset are correctly classified as class 27. The workload classification model predicts labels of two instances as 27 with a probability 0.99. We show Top-5 super features based on the weights which refer to the amount of attribution each feature gives to the prediction. Super feature 13 gets the highest weight from both instances indicating that access patterns of super feature 13 affect the prediction the most. Super features 2 and 11 commonly have high ranks in both instances while super feature 0 gives a negative impact on the predictions of two instances. From the results, we find that cell-level super features are important for the workload 27. Fig 7 shows an example of interpretations using two instances from class 9 in the SEC-seq dataset. We report the attribution values of classifying the instances to class 9. Super feature 2 gets the highest weight from both instances which means the corresponding n-gram sequences have important patterns for the workload 9. We find several negative weights from the interpretation of instance 4 which is misclassified as class 8. Note that super features 1, 11, and 3 restrain the instance 4 from being classified into class 9.

**Fig 8:**
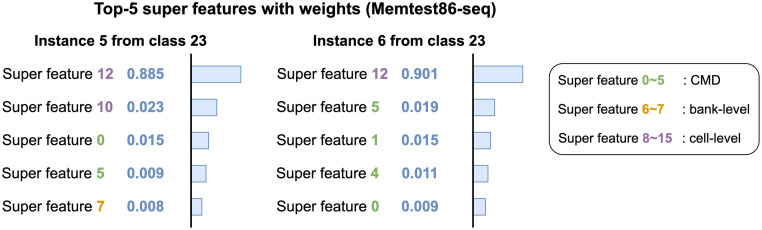
Interpretation of two instances for class 23 from Memtest86-seq dataset. Both instances are correctly classified as class 23. A cell-level super feature 12 and CMD super features commonly give a positive impact on the results.

**Fig 9:**
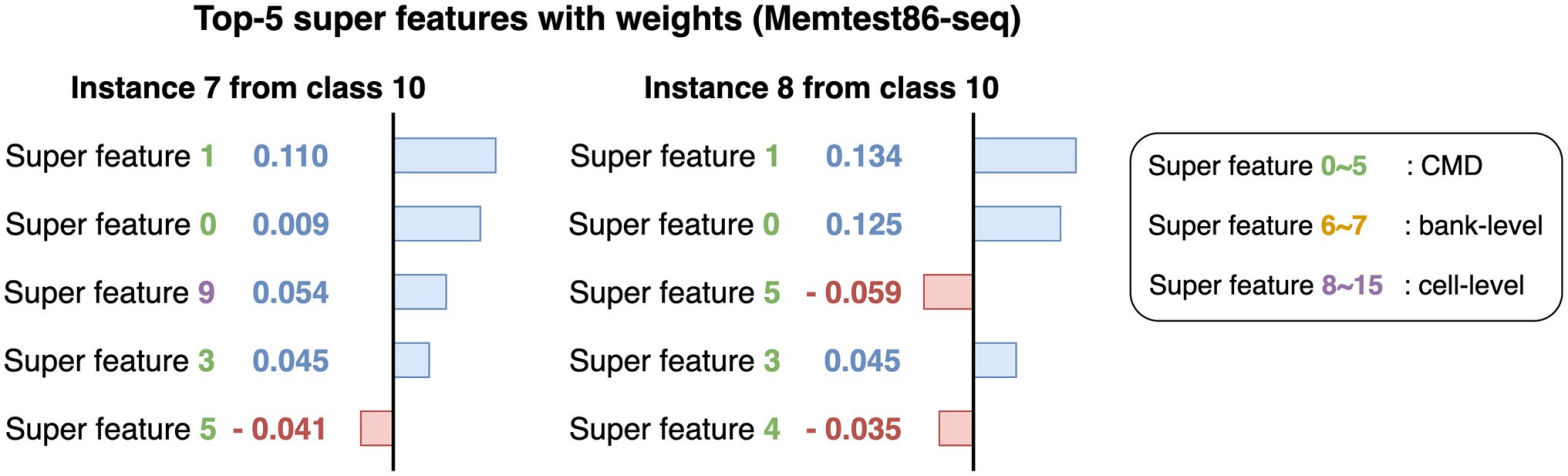
Interpretation of two instances for class 10 from Memtest86-seq dataset. Instance 7 is correctly classified as class 10 while instance 8 is misclassified as class 9. CMD super features 1, 0, and 3 affect the predictions the most. Note that instance 8 contains more negative weights than instance 7 leading to misclassification.

Fig 8 shows the interpretation results of two instances that belong to class 23 in the Memtest86-seq dataset. Super features 12, 0, and 5 commonly appear in Top-5 super features from both instances. The results show that CMD super features and a cell-level super feature contain important patterns of class 23 giving a positive impact on the predictions. Fig 9 shows the results of two instances from class 10 in the Memtest86-seq dataset. Instance 8 is misclassified as class 9; thus the interpretation of the instance 8 on class 10 has more negative weights than that in the instance 7. CMD super features 1, 0, and 3 have the highest weights in both instances while super feature 5 restrains two instances from being classified into class 10. Note that super feature 4 gets negative weight on the instance 8 which leads to misclassification. Overall, the results of interpretations tell us that bank-level super features do not affect the predictions as much as the others and certain types of super features influence each workload.

**Fig 10:**
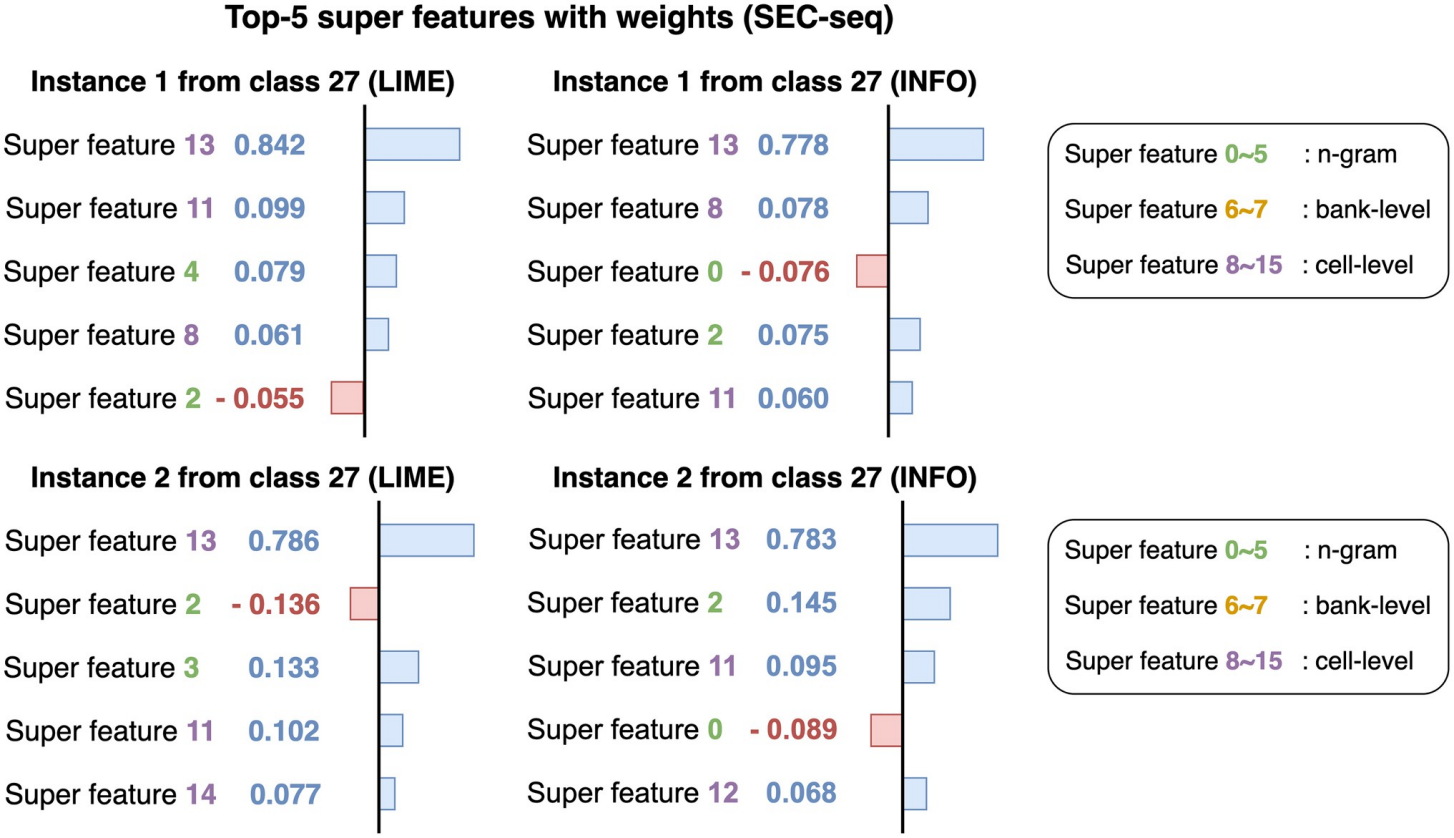
Comparison of interpreting two instances from SEC-seq dataset using LIME and INFO. Both instances are correctly classified as class 27. Note that super features of two instances generated from LIME are different resulting in inconsistent interpretations while INFO utilizes the same super features over all instances.

Fig 10 compares the interpreted results of two instances using LIME and INFO. For interpretations of LIME, super features 13 and 11 are commonly ranked as Top-5 super features from both instances but it does not mean that the features within super features 13 and 11 are crucial for class 27. Since LIME generates super features per instance, super features used to explain the predictions of two instances contain different features resulting in inconsistent explanations. On the other hand, INFO utilizes global super features commonly defined for the entire instances. Thus, we know that super feature 13 contains the most important patterns for class 27 while super feature 0 gives a negative influence on classifying instances to class 27. Table 3 shows an example of CMD super features using LIME and INFO. In LIME, we use two instances from class 27 and 9 in the SEC-seq dataset to show the clustering results denoted as LIME (27) and LIME (9), respectively. N-gram sequences within super feature 0 from LIME (27) commonly have 31133 while n-grams of super feature 0 from LIME (9) have 161313 in common. In contrast, INFO generates global super features that are consistently used for all instances. The results show that INFO enables both instance-wise and class-wise interpretations.

**Table 3:** Example of CMD super features generated by LIME and INFO. LIME (27) and LIME (9) show the clustering results of two instances from class 27 and class 9 in the SEC-seq dataset, respectively. INFO makes the same super features over all instances while LIME gives inconsistent super features.

Method	Super feature ID	Example of n-gram sequences
LIME (27)	Super feature 0	**31133**13, 31**31133**, 113**31133**113
	Super feature 1	**511111**3, 1**511111**3333, 1**511111**31333551
LIME (9)	Super feature 0	**161313**1, 51515**161313**, 5**161313**
	Super feature 1	**511551**1, 33115**511551**, 115**511551**15
**INFO**	Super feature 0	**13131313**151, 5**13131313**13, 15**13131313**1
	Super feature 1	**113131**3, 1**113131**, 5111**113131**3

### Qualitative analysis of clustering

**Fig 11:**
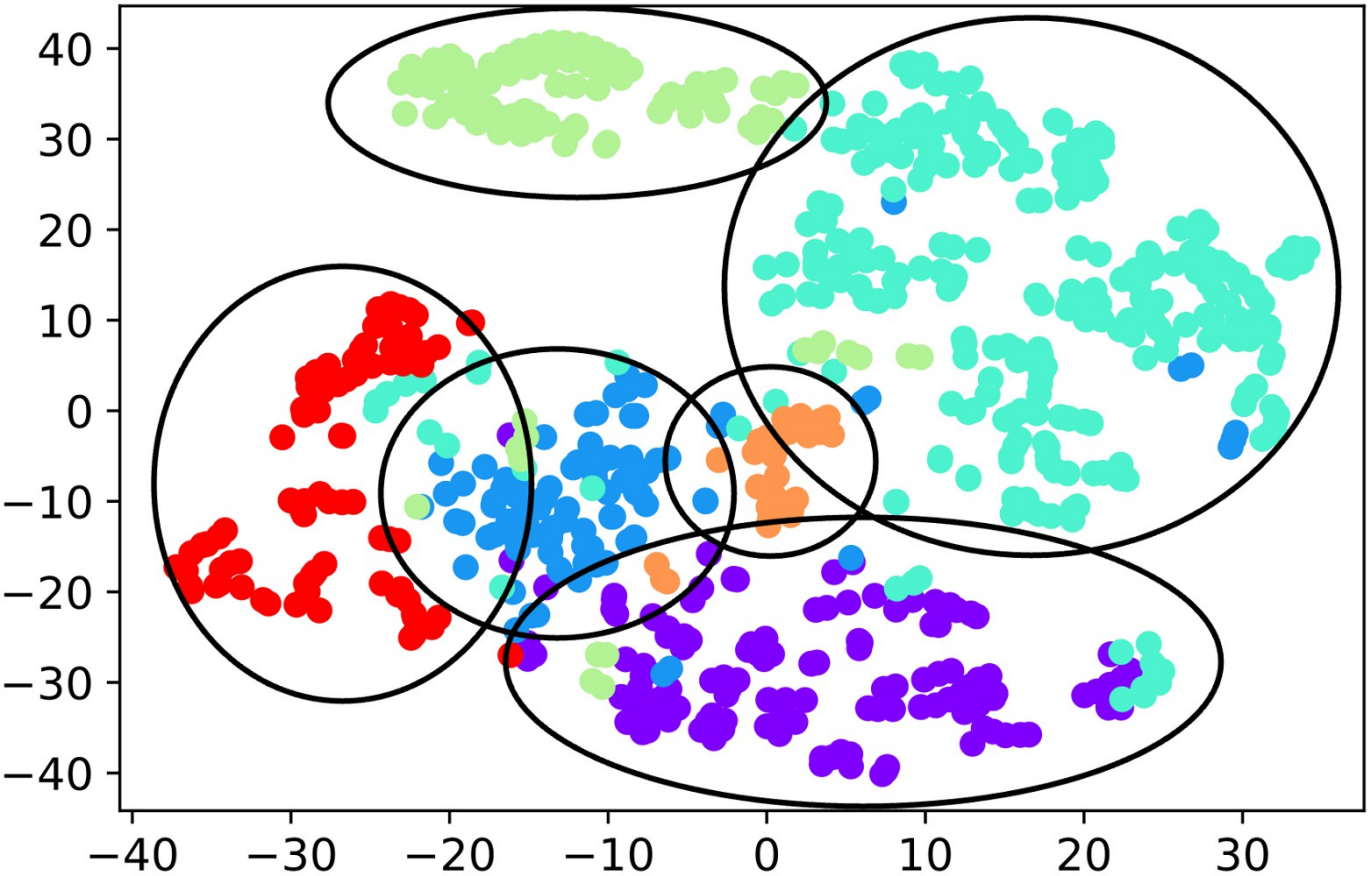
Visualization of CMD super features. We cluster n-gram sequences based on a variant of Jaccard similarity.

We provide qualitative analysis for the clustering of n-gram features. We construct the similarity matrix for hierarchical clustering using the interpretability-friendly similarity we proposed. To analyze the clustering result, we visualize clusters using t-SNE, a dimensionality reduction strategy based on the similarity matrix. Fig 11 shows the visualization result where each color represents a super feature. Note that the super features are well separated. We also report an example of CMD super features in Table 4. All the super features contain recurring subsequences. The results show that super features are well-captured based on the similarity measure.

**Table 4:** Example of CMD super features. The bold text represents the repetitive patterns within each cluster.

Super feature ID	Example of n-gram sequences
Super feature 0	**13131313**151, 5**13131313**13, 15**13131313**1
Super feature 1	**113131**3, 1**113131**, 5111**113131**3
Super feature 2	131313131515151, 1**111333**, **111333**3
Super feature 3	**3311**313, 31311**3311**33, 131**3311**
Super feature 4	**51113133315**, 151**51113133315**1, 51**51113133315**11
Super feature 5	515151**51111**, 5151**51111**333315, **51111**333315

## Related works

We describe related works on interpretable models.

### Local explanation model

Locally interpretable models take only one instance and supply the best answer fitted to a given instance. CAM [3] provides visualized explanations by modifying the pooling layer of convolutional neural networks in the image domain. Selvaraju et al. [4] introduce Grad-CAM, a generalized version of CAM. LIME [2] proposes super-pixels to interpret an image classification model. SHAP [5] proposes a generalized framework of interpretable models based on the concept of game theory. MAPLE [6] gives local explanations by fitting the model directly to a given instance and estimates the prediction of a black box model. Adebayo et al. [7] give local explanations by re-initializing the weights of deep neural networks and introduce a similarity measure between the explanations based on the weights. Ghalebikesabi et al. [8] propose Neighbourhood Shapley values which improve the local interpretability of Shapley values by weighting features based on a distance metric. Graziani et al. [9] apply LIME to clinical decision-making. DLIME [10] changes random-based perturbation of LIME to a deterministic version using agglomerative hierarchical clustering and k-nearest neighbor. LUC-Locator [11] finds optimal LUCs which are sets of input words sufficient to justify the prediction. SIDU [12] shows visual explanations of a given image addressing a salient region localization issue. ExSum [13] proposes a mathematical method to improve the quality of local explanations. LIMREF [14] provides rule-based local explanations for a particular forecast given by the global forecasting model. However, none of the previous local explanation methods are developed for workload classification. Furthermore, they are limited to local explanations, without giving global explanations. We propose INFO which provides global explanations for workload classification results by exploiting global super features.

### Global explanation model

If an interpretable model explains the predictions globally, it makes a consistent response to the entire test set. Since intrinsic models (e.g., decision tree) have interpretability itself, their explanations are viewed as global. Sushil et al. [15] formalize the explanations to generate them globally. GALE [16] proposes an aggregation strategy to provide global explanations. MUSE [17] generates global explanations by defining subspaces of features specified by user interest and making decisions through a black box model. GLocalX [18] attaches an interpretable layer to a black box model and iteratively gathers local explanations to generate global explanations. Jacovi et al. [19] propose contrastive explanations which capture the difference between two representation vectors for text data. CIE [20] generates confident itemsets corresponding to a specific class and provides both instance-wise and class-wise explanations. Asano et al. [21] define two hypersphere sets for a global surrogate model to show high recall and high precision, respectively. Previous approaches for global explanations are proposed only in the text domain. In this work, we propose an interpretability-friendly similarity to generate global super features for workload subsequences.

## Conclusion

In this paper, we propose INFO, a globally explainable model which provides fast and accurate interpretations for workload classification. INFO provides consistent interpretations for all workload subsequences through global super features. To generate super features, we propose an interpretability-friendly similarity measure between the raw features for workload classification based on a variant of Jaccard similarity. We generate 3 different types of super features for interpretation: CMD, bank-level, and cell-level super features. For CMD super features, we compute the similarity between n-gram sequences and hierarchically cluster the sequences based on the similarity. We also follow the inherent hierarchy of bank-level fields, i.e., rank, bank group, and bank fields, setting each rank as a bank-level super feature. Since the address field indicates an exact location of command operation in a particular bank, we divide each bank into memory blocks and group them considering the locality. Each group is used as a cell-level super feature. INFO trains a linear model to approximate a workload classifier and globally explains the classifier utilizing the super features. Experiments show that the INFO improves computational efficiency while giving accurate and consistent interpretations which are faithful to the workload classification model.
